# Investigating a Potential Map of PM_2.5_ Air Pollution and Risk for Tourist Attractions in Hsinchu County, Taiwan

**DOI:** 10.3390/ijerph17228691

**Published:** 2020-11-23

**Authors:** Yuan-Chien Lin, Hua-San Shih, Chun-Yeh Lai, Jen-Kuo Tai

**Affiliations:** 1 Department of Civil Engineering, National Central University, Taoyuan 32001, Taiwan; misscindy555@gmail.com (H.-S.S.); harry2070@hotmail.com (C.-Y.L.); 0326@hchg.gov.tw (J.-K.T.); 2 Research Center for Hazard Mitigation and Prevention, National Central University, Taoyuan 32001, Taiwan; 3 Fire Bureau, Hsinchu County Government, Hsinchu County 30295, Taiwan

**Keywords:** air pollution potential map, PM_2.5_, spatial analysis, tourist attractions, risk analysis, GIS

## Abstract

In the past few years, human health risks caused by fine particulate matters (PM_2.5_) and other air pollutants have gradually received attention. According to the Disaster Prevention and Protection Act of Taiwan’s Government enforced in 2017, “suspended particulate matter” has officially been acknowledged as a disaster-causing hazard. The long-term exposure to high concentrations of air pollutants negatively affects the health of citizens. Therefore, the precise determination of the spatial long-term distribution of hazardous high-level air pollutants can help protect the health and safety of residents. The analysis of spatial information of disaster potentials is an important measure for assessing the risks of possible hazards. However, the spatial disaster-potential characteristics of air pollution have not been comprehensively studied. In addition, the development of air pollution potential maps of various regions would provide valuable information. In this study, Hsinchu County was chosen as an example. In the spatial data analysis, historical PM_2.5_ concentration data from the Taiwan Environmental Protection Administration (TWEPA) were used to analyze and estimate spatially the air pollution risk potential of PM_2.5_ in Hsinchu based on a geographic information system (GIS)-based radial basis function (RBF) spatial interpolation method. The probability that PM_2.5_ concentrations exceed a standard value was analyzed with the exceedance probability method; in addition, the air pollution risk levels of tourist attractions in Hsinchu County were determined. The results show that the air pollution risk levels of the different seasons are quite different. The most severe air pollution levels usually occur in spring and winter, whereas summer exhibits the best air quality. Xinfeng and Hukou Townships have the highest potential for air pollution episodes in Hsinchu County (approximately 18%). Hukou Old Street, which is one of the most important tourist attractions, has a relatively high air pollution risk. The analysis results of this study can be directly applied to other countries worldwide to provide references for tourists, tourism resource management, and air quality management; in addition, the results provide important information on the long-term health risks for local residents in the study area.

## 1. Introduction

Air pollution is a topic of concern worldwide; it affects the atmospheric and ecological environment and poses a serious threat to the health of humans. Owing to the rapid development of the modern industrial society, global climate change, and increasing environmental awareness of people, air pollution has received increasing attention. According to the Disaster Prevention and Protection Act of Taiwan’s Government enforced on 22 November 2017, “suspended particulate matter” has officially been acknowledged as a disaster-causing hazard.

Haze is caused by extremely small dry particles in the air, which impair visibility. Suspended particulate matter can be classified according to the particle diameter. Particles with sizes of less than 10 μm are PM_10_, and those with sizes of less than 2.5 μm are PM_2.5_. The different particle sizes have different effects on the human body; PM_2.5_ is smaller than PM_10_ and can therefore penetrate the human cilia and mucus, reach the bronchi and alveoli and then the walls of the bronchioles, and finally interfere with the gas exchange in the lungs. In addition, PM_2.5_ is more easily suspend in air, does not settle easily, and interacts with other air pollutants [1,2]. Once inhaled by a human, PM_2.5_ can reach the depth of the lungs and even penetrate the alveoli and enter the cardiovascular system. As blood circulates throughout the entire body, the harm to human health and ecology is more severe than that from other suspended particulate matter [3,4,5,6]. Many researchers have further pointed out that airborne fine particulate matter can directly or indirectly lead to chronic respiratory diseases, cardiovascular diseases, cancer, neurotoxicity, and even dementia diseases [7,8,9,10,11]. In addition, the long-term exposure to high-concentrations of air pollutants is even more harmful [12,13,14]. Therefore, analyzing the long-term spatial distributions of air pollution hazards (particularly PM_2.5_) will provide valuable information for protecting the health and safety of residents.

Over the past few years, China has repeatedly experienced extremely hazardous PM_2.5_ concentrations [15,16]. For example, on 19 October 2016, 11 provinces in China were severely affected by air pollutants. Moreover, many cities in western Taiwan are affected by both transboundary and local pollutants, and their air quality is very poor. As Taiwan’s geographical location is close to the southeast of China, it is also the main route for the cold high pressure traveling from China in winter; the transboundary pollutants from China may affect Taiwan’s air quality and the atmospheric circulation. In addition, local or regional sources of pollutants, such as transportation vehicles and factories, produce airborne particulate matter [17,18,19].

The spatial analysis of disaster potentials is a very important part of risk assessments. As assessing the risk of hazards is crucial for a timely evacuation, the analysis of disaster potentials has become very common. The Water Resources Agency of the Ministry of Economic Affairs of Taiwan and researchers have published and applied several generations of flood potential maps for many years [20,21]. In addition, in the Taiwan Central Geological Survey, researchers developed and applied soil liquefaction potential maps [22,23,24]. However, potential disasters caused by suspended particulate matter and the spatial characteristics of air pollution in the past have not been comprehensively investigated; i.e., no potential map of PM_2.5_ has been drawn before. In addition, a pollution potential map of various regions would provide valuable information.

Tourist attractions are important gathering places for people, particularly on holidays. Most visitors wish to relax and expect high air quality. Many researchers have studied the relationship between areas of interest and air quality; in particular, they have investigated the integration of low-cost air quality monitoring Internet of Things systems and air quality big data models [25,26,27,28,29,30]. Over the past few years, some Chinese researchers have analyzed the air pollution characteristics of certain specific tourist attractions [31,32]. However, the relationship between the overall tourist attractions and air quality has not been studied.

Hsinchu County in Taiwan has diversified industry, with equal emphasis on agriculture, industry, technology, businesses, and leisure tourism. In addition, Hsinchu County is adjacent to Hsinchu City and Hsinchu Science Park. The population and industry are developing rapidly, and large numbers of people enter Hsinchu County’s major tourism and recreation areas every holiday season. Therefore, high air quality around tourist attractions is very important. A previous study of the characteristics of air pollutants in Hsinchu has shown that the PM_2.5_, total PAHs (Polycyclic Aromatic Hydrocarbons), and BaPeq (benzo(a)pyrene equivalent) mass concentrations during the seasons had the following order: winter > autumn > spring > summer with significant seasonal variations [33]. Some early studies focused on the impacts of the large and dense high-tech industries in the Hsinchu Science Park on health and the environment [34,35,36]; in addition, the researchers considered the emissions of toxic compounds such as VOCs (Volatile Organic Compounds) and arsenical emissions; however, there have been few relevant studies in the past decade.

Therefore, the objective of this study was to investigate the exposure risks of tourist attractions based on the potential map of PM_2.5_ calculated by the exceedance probability and spatial estimation methods. In this study, the Hsinchu County area was taken as an example. Historical data of PM_2.5_ concentrations from the Taiwan Environmental Protection Administration (TWEPA) were used to analyze spatially the air pollution hazard potential of PM_2.5_ concentrations in Hsinchu County based on geographic information system (GIS) statistics. The potential threat of PM_2.5_ concentrations exceeding a certain standard was spatially investigated with the exceedance probability method; furthermore, the air pollution risk levels of areas with tourist attractions in Hsinchu County were determined. The analysis results of this study can be directly applied to other countries worldwide; they provide references for tourists, tourism resource management, and air quality management, and important information on the long-term health risks for local residents in the study area.

## 2. Materials and Methods

### 2.1. Study Area

**Figure 1:**
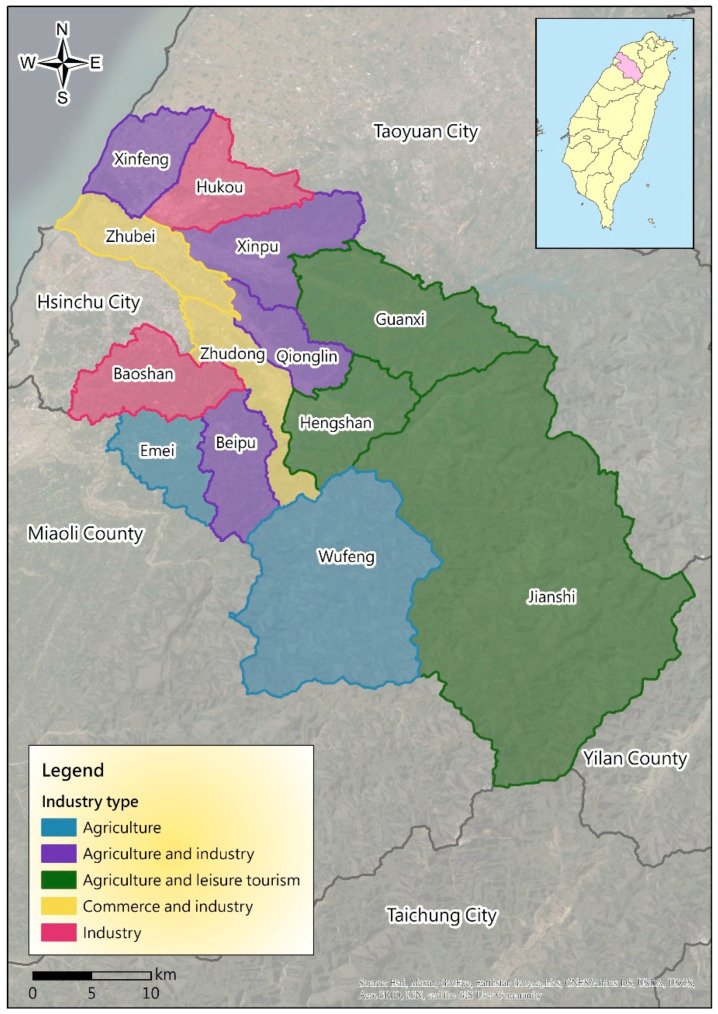
Main industry types of 13 townships and cities in Hsinchu County.

The terrain of Hsinchu County is mainly composed of flat land, hills, and mountains. There are 13 administrative districts (towns and cities), and its development industries are diverse; they can be mainly classified into agriculture, industry (including science and high technology), commerce, and leisure tourism (Figure 1). Zhubei City is an important town in terms of commerce, economics, and politics; its industries develop high-tech electronics (such as in the Taiyuan Science and Technology Park). The inflow of industrial capital into Zhudong town comprises real estate capital and high-tech manufacturing capital. Like those of Zhubei City, its industries comprise mainly commerce and industry (such as the Industrial Technology Research Institute). The Hukou and Baoshan Townships have the most developed industries and are the production bases for the technology and manufacturing industries, such as the Hsinchu Industrial Park of the Industrial Development Bureau, the Ministry of Economic Affairs in Hukou Township, and high-tech companies such as Taiwan Semiconductor Manufacturing Company and other high-tech factories in Baoshan Township. Baoshan Reservoir and Baoshan Second Reservoir are important water resources for the Hsinchu Science Park. Moreover, the Emei and Wufeng Townships focus on agriculture (tea, oranges, peaches, and sweet persimmons). The Xinfeng, Xinpu, Qionglin, and Beipu Townships exhibit agricultural activities and the establishment of regional industrial zones for the industrial development. Guanxi town, Jianshi Township, and Hengshan Township focus mainly on agriculture and the development of tourism and leisure industries (such as Guanxi Grass, Neiwan Old Street, orchard sightseeing, and visits to the Taiwanese aboriginal people).

To minimize the impacts of disasters, the disaster characteristics of local key industries are studied based on disaster potential data. The results should be sent to the local governmental agencies and key industries (such as the industrial and agricultural management units) in Hsinchu County as an important reference for disaster prevention. More importantly, improvements in areas with higher risks should be prioritized. The main disaster types faced in Hsinchu County can be roughly distinguished according to the topography. The administrative areas on flat land, such as the Zhudong, Hukou, and Xinpu Townships, may experience floods and droughts, and the mountainous administrative areas, such as the Jianshi and Wufeng Townships, can predominantly suffer from landslides or mudflows; in addition, the area close to the sea may face tsunamis. Owing to the development of industrial areas, the Hukou, Baoshan, and Qionglin Townships may suffer man-made disasters caused by toxic chemicals and air pollutants. The industrial characteristics and major and minor risks in the 13 towns and cities in Hsinchu County are summarized in Table 1.

### 2.2. Framework of Risk Analysis

**Figure 2:**
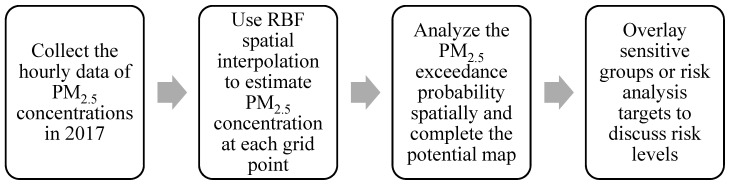
Flow chart of the construction of an air pollution potential map and risk analysis.

The potential refers to the frequency or probability of the occurrence of disasters in an area; the determined potential can be used as a reference for future risk assessments. In this study, the hourly data of PM_2.5_ concentrations measured by the TWEPA in Taiwan in 2017 were used, and spatial interpolation was applied to estimate the hourly PM_2.5_ concentration of each grid point in the county. Subsequently, the probability that the PM_2.5_ concentration of each grid point exceeds the standard value statistically was calculated. The air quality index that corresponds to the unhealthy PM_2.5_ concentration for sensitive groups (35.4 μg/m^3^) was used as the concentration standard. This probability can be represented based on the exceedance probability of older data, which represents the spatial distribution of the potential of PM_2.5_. The analysis process is shown in Figure 2.

After determining the spatial distribution of the air pollution potentials, the air pollution risk levels in various tourist areas in Hsinchu County were examined. As shown in Figure 2, the PM_2.5_ concentrations are based on data from the TWEPA’s Taiwan-wide air quality-monitoring stations from 2017; in addition, radial basis function (RBF) spatial interpolation was used to estimate the grid-like PM_2.5_ concentrations in the Hsinchu County area, and the exceedance probability method was applied to calculate the probability that the PM_2.5_ concentration of each grid point exceeds the standard. Finally, the potential air pollution risks in the areas of the major tourist attractions in Hsinchu County were examined. The PM_2.5_ concentration standard used in this study is based on the air quality index, which considers six levels: good, normal, unhealthy for sensitive groups, unhealthy for all people, very unhealthy, and hazardous. When the “unhealthy for sensitive groups” degree has been reached, it is generally recommended that residents reduce outdoor activities and prolonged vigorous exercise. Therefore, the PM_2.5_ concentration standard (35.4 μg/m^3^) corresponding to the “unhealthy air quality for sensitive groups” degree was used as the threshold. In this study, the analysis results of the probability that the PM_2.5_ concentration exceeds the standard were classified into eight levels. In addition, most areas of the Jianshi and Wufeng Townships are too far from the TWEPA’s air quality monitoring station (15 km from the monitoring station) and mainly in high mountainous terrain; thus, they were not included in the calculations.

### 2.3. Data Collection

First, the hourly PM_2.5_ concentrations collected by 76 air quality-monitoring stations of the TWEPA in Taiwan in 2017 were collected. The 327 datasets from areas with tourist attractions originate from the official open data website of the Hsinchu County Government, which were collected in 2019 (https://www.hsinchu.gov.tw/OpenDataDetail.aspx?n=902&s=272).

### 2.4. Spatial Analysis of Data

The PM_2.5_ concentrations throughout Taiwan were estimated with the data from the monitoring stations and RBF spatial interpolation method [37,38,39]. The RBF interpolation is one of the most precise interpolation methods. The interpolation function must pass through the observation value of each station and generate a smooth surface. RBF interpolation is a mesh-free method, constructing high-order accurate interpolants of unstructured data. It takes the form of a weighted sum of radial basis functions. In addition, the RBF interpolation method uses a symmetric function centered at each observation point and calculates the change in the distance from the observation point to obtain the weight of each function:

(1) $$\left[\begin{array}{ccc} \varphi \left(∥x_{0}-x_{0}∥\right) & \cdots & \varphi \left(∥x_{n}-x_{0}∥\right)\\ \vdots & \ddots & \vdots \\ \varphi \left(∥x_{0}-x_{n}∥\right) & \cdots & \varphi (∥x_{n}-x_{n}∥) \end{array}\right]\left[\begin{array}{c} w_{0}\\ \vdots \\ w_{n} \end{array}\right]=\left[\begin{array}{c} f\left(x_{0}\right)\\ \vdots \\ f\left(x_{n}\right) \end{array}\right]$$

where φ is a centrosymmetric function and $w_{n}$ the weight of each function; the interpolation function $f\left(x_{n}\right)$ can be obtained by solving the equations.

The RBF interpolation method has a good effect on flat surfaces (for concentration diffusion, for instance). In this study, Taiwan was divided into approximately 32,000 grid points, the hourly PM_2.5_ concentration of each grid point was estimated with the RBF interpolation method, and the probabilities that the grid points exceed the concentration standard were determined; and finally, we cut and selected the study area of Hsinchu County; ESRI ArcGIS was used to calculate and draw the exceedance probability map. In this study, the exceedance probability of the hourly air pollution concentration was defined as the probability that the hourly data (of an entire year) exceed a certain concentration standard. The air quality index that corresponds to the unhealthy PM_2.5_ concentration for sensitive groups (35.4 μg/m^3^) was used as the concentration standard:

(2) $$P_{E}=\frac{N_{E}}{N_{all}}$$

where $P_{E}$ is the exceedance probability, $N_{E}$ the number of times in which the hourly data exceed a certain concentration standard in one year, and $N_{all}$ the total number of hourly data of one year. The research data were analyzed with Python and ESRI ArcGIS.

## 3. Results

### 3.1. Analysis of Air Pollution Potential

**Figure 3:**
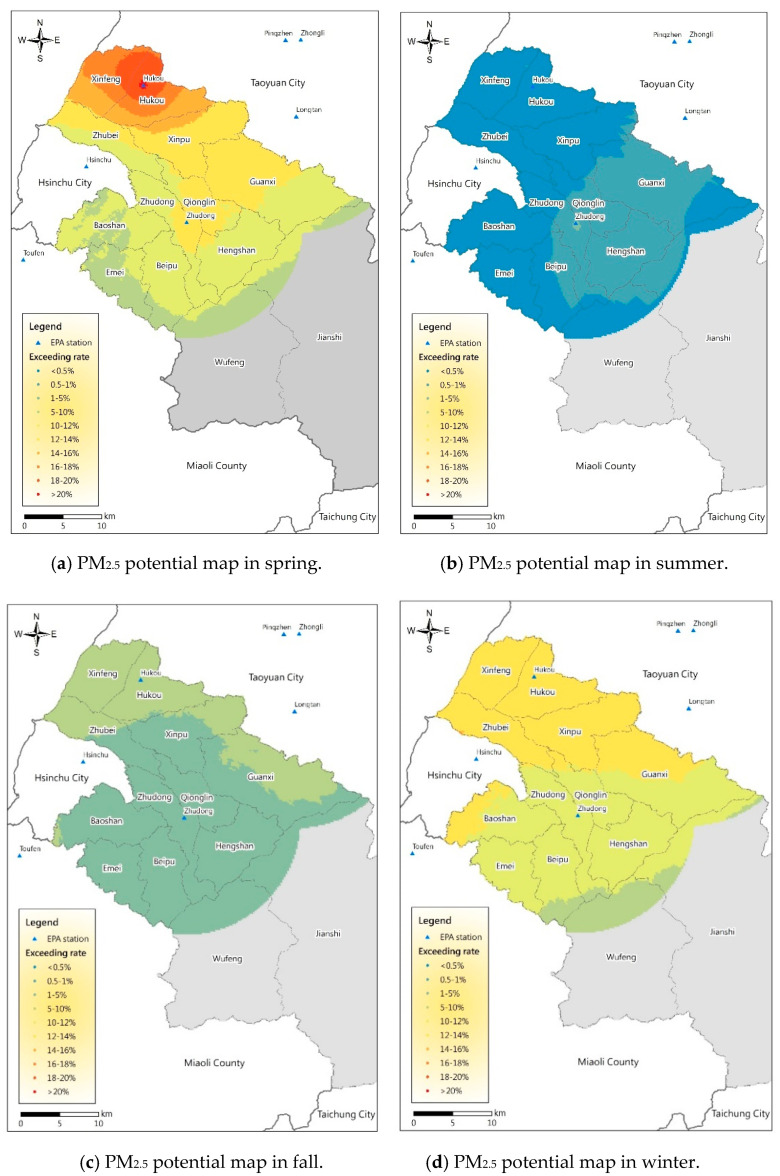
Distribution of PM_2.5_ potential in the study area in Hsinchu County in different seasons. Overall, 76 air quality-monitoring stations of the Taiwan Environmental Protection Administration (TWEPA) across the whole of Taiwan were used for spatial estimation, and we extracted the region of Hsinchu County for further analysis. (**a**) PM2.5 potential map in spring. (**b**) PM2.5 potential map in summer. (**c**) PM2.5 potential map in fall. (**d**) PM2.5 potential map in winter.

**Figure 4:**
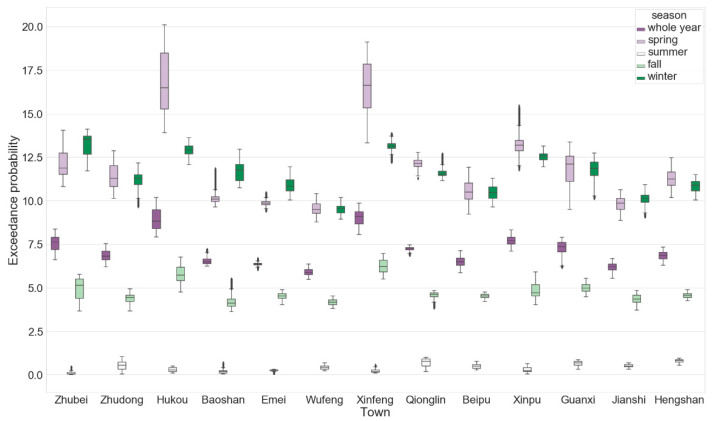
Boxplot of exceedance probabilities of 13 townships and cities in Hsinchu County.

The PM_2.5_ concentration is greatly affected by meteorological factors; therefore, the data were investigated according to the different seasons (spring: March–May; summer: June–August; autumn: September–November; winter: December–February). The results are shown in Figure 3. The gray area is too far from the air quality station and was therefore excluded. The analysis results show that the pollution potential in spring (Figure 3a) and winter (Figure 3d) is higher; the probability that the standard concentration in all towns and cities is exceeded is 9.5%, particularly in spring when the Xinfeng and Hukou Townships have probabilities of more than 18%; the probability decreases from the northwest plain area to the southeast mountainous area. The pollution potential in summer and autumn is relatively low; the probability that the standard is exceeded in autumn is generally only approximately 5%. The potential in the northern area of Hsinchu County adjacent to Taoyuan City is higher. In summer, the probability does not exceed 1%, and the probability of pollution in the area near Zhudong Station is slightly higher. Figure 4 and Table 2 show the detailed boxplots and basic statistics of the exceedance probabilities of the 13 townships and cities in Hsinchu County, respectively.

### 3.2. Risk Analysis of Areas with Tourist Attractions

**Figure 5:**
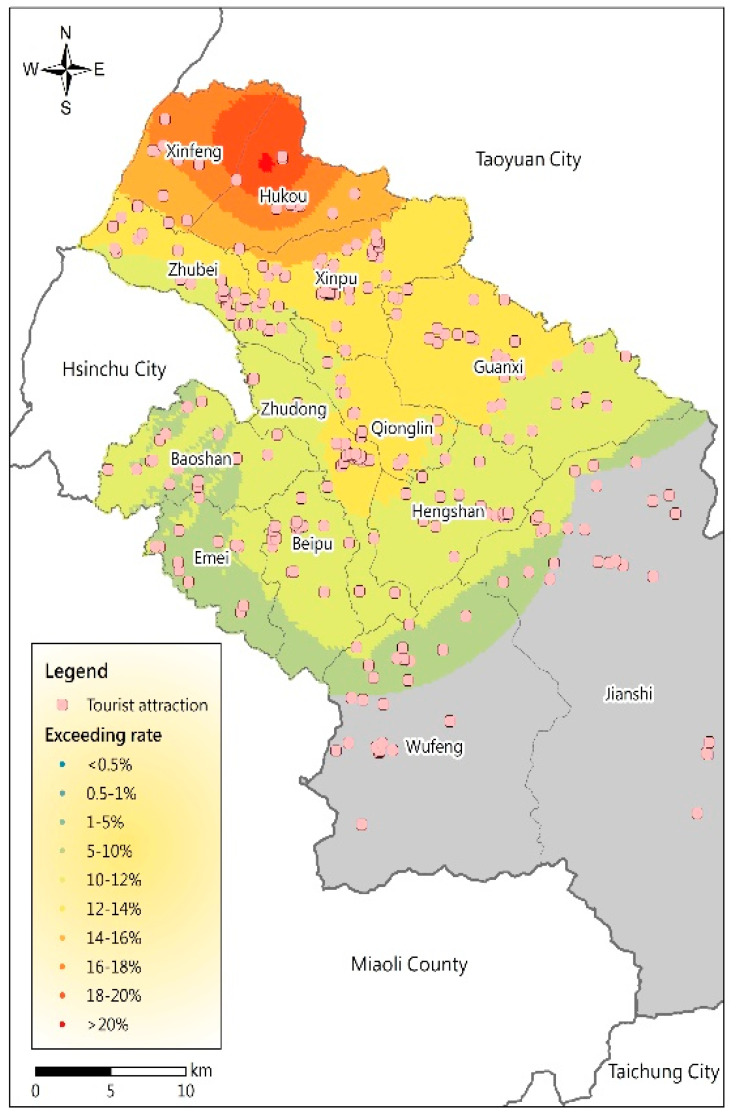
Distribution of PM_2.5_ potential in tourist areas in Hsinchu County in spring in 2017.

The spatial distribution map of the PM_2.5_ potential was overlaid on a map of the various tourist areas in Hsinchu County; the most severe spring PM_2.5_ potential was chosen, as shown in Figure 5, Table 3 and Table 4. The results show that the probability that the standard is exceeded is greater than 18%; the areas with the most severe air pollution potential level (level 6) have three important tourist attractions: the Caixiang Trail, Xiansheng Temple, and Hukou Armored New Village (Village B). The areas of level 5 (16% to 18% chance of exceeding the standard) and level 4 (14% to 16% chance of exceeding the standard) potential—slightly higher potential—have 11 and 7 tourist attractions, respectively. The 11 tourist attractions with level 5 potential are Rongyuanpu Farm, Laohukou Catholic Church Cultural Center, Renhe Trail, Yao Art Street and Bicycle Taro, Hanqing Trail, Hukou Old Street, Xinfeng Sanyuan Temple, Yongning Temple, Chifu Wangye Temple, Hongmaogang Ecological Recreation Area, and Xinfengpuyuan Temple. Another 114 tourist areas are at level 3 (exceeding rates of 12% to 14%), and 124 tourist areas are at level 2 (exceeding rates of 10% to 12%); these locations still exhibit rates greater than 10% in spring (Table A1). These areas encounter a higher risk of air pollution with excessive PM_2.5_ concentrations. The highest air pollution potentials of the tourist attractions with levels 5 and 6 in Hsinchu County are shown in Table 4; they are located in the Hukou and Xinfeng Townships. As many tourist areas in Hsinchu County are located in hilly or mountainous areas, they are less exposed to PM_2.5_. Only the scenic spots in the Hukou and Xinfeng Townships experience relatively high PM_2.5_ concentrations. The detailed PM_2.5_ air pollution potential of each tourist attraction in Hsinchu County is shown in Appendix A.

### 3.3. Analysis of Population Density and Air Pollution Exposure Risk

**Figure 6:**
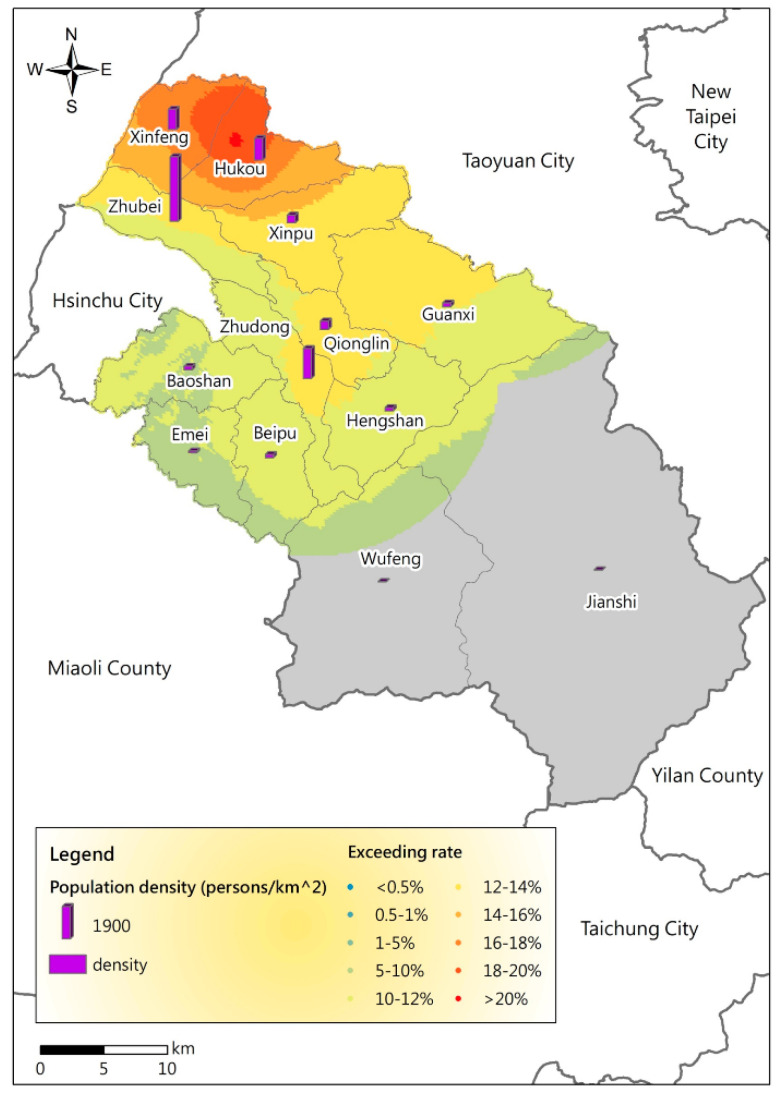
PM_2.5_ potential distribution and population density of each township in Hsinchu County.

**Figure 7:**
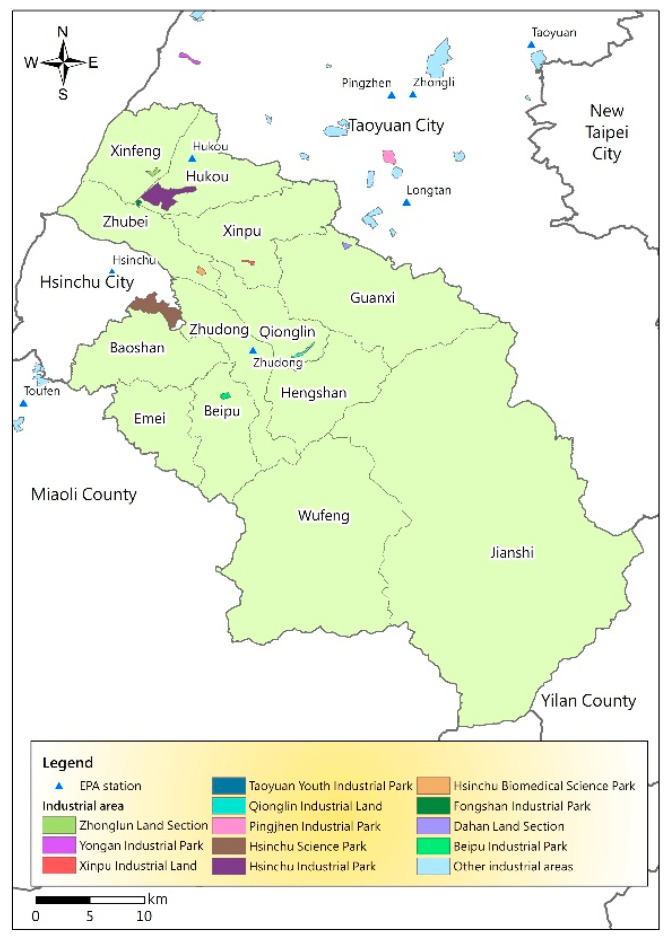
Map of industrial areas and air quality stations.

Moreover, the PM_2.5_ potential spatial distribution map was investigated based on the population density of each township in Hsinchu County (Table 5) to analyze the long-term air pollution exposure risks for residents. According to Figure 6, the population density is correlated with the PM_2.5_ potential distribution. The Pearson correlation coefficient between the PM_2.5_ potential and population density in towns throughout the year is 0.44. If it is explored according to the season, the correlation coefficients between the PM_2.5_ potential and population density in spring, summer, autumn, and winter are 0.36, −0.46, 0.34, and 0.64, respectively. Zhubei City (3885.10 persons per square kilometer), Zhudong town (1811.10 persons per square kilometer), Hukou Township (1325.41 persons per square kilometer), and Xinfeng Township (1226.25 persons per square kilometer) have higher population densities than the remaining areas and therefore higher PM_2.5_ potentials. A high population density reflects the degree of development and traffic in the city. According to Figure 7, the main industrial areas of Hsinchu County are mostly concentrated in these towns and villages and the main source of pollution. Owing to the prevailing northeast monsoon conditions in winter, these areas have higher pollution risks. Although many tourist attractions are not located in the areas with high air pollution potentials, many residents live in areas with relatively high air pollution potentials for a long time.

## 4. Discussion

The change in and accumulation, diffusion, and transmission of PM_2.5_ concentrations are greatly affected by the meteorological conditions or weather patterns [40,41,42]. The analysis results of the air pollution potentials in Figure 3 are consistent with the general air pollution season in Taiwan (winter and spring). The main reason is that the main prevailing wind in Taiwan in winter and spring is the northeast monsoon; thus, the western half is not affected because of the mountains. The leeward places are likely to experience accumulations of pollutants, particularly central and southwestern Taiwan [41,43,44]. Furthermore, the northeast monsoon tends to bring foreign pollutants from west China into this area [45]. Therefore, the Xinfeng and Hukou areas in Hsinchu County have the highest pollution potentials in winter and spring. In addition, Hsinchu Industrial Park lies in the Xinfeng and Hukou area, and the northern region is close to major stationary pollution sources, such as Taoyuan Youth Industrial Park, Pingjhen Industrial Park, and Yongan Industrial Park (Figure 7). Zhubei City and Hsinchu Science Park in the south are densely populated areas with long-term traffic congestion and are the main sources of mobile pollution in Hsinchu County and Hsinchu City [34,35,36,46]. Both spring and winter are high-pollution seasons, but spring exhibits more evident pollution sources (Figure 3).

In order to further compare the PM_2.5_ potential distribution in different years, in addition to Figure 5 showing 2017, Figure 8 shows the dynamic distributions of PM_2.5_ potential in tourist areas in Hsinchu County in spring in 2018 and 2019. They show spatial distributions similar to 2017, and Xinfeng and Hukou also have the highest potential. However, it is obvious that the overall probability of PM_2.5_ exceeding the standard has been declining in the entire region in recent years. In addition to the influences of meteorological conditions in different years, it may be due to the implementation of government policies and the increase in people’s awareness of environmental protection.

Moreover, Xinpu, Guanxi, Qionglin, Baoshan, Emei, and Beipu are dominated by hilly land; this less densely populated area exhibits agricultural, industrial, and touristic activities; thus, the air quality is evidently better than in other areas in all seasons. The Hengshan, Jianshi, and Wufeng Townships have mostly mountainous terrain, and the populations are sparser; consequently, they have the best air quality. In addition, because the west side of Hsinchu is adjacent to the sea and the east side exhibits mostly hilly terrain, the topographical effect is affected by the prevailing wind and major sources of emissions in the air pollution season [47]. Therefore, air pollutants in Hsinchu accumulate easily in the relatively flat plains, such as in Xinfeng and Hukou, which is consistent with the results of this study. Some researchers have investigated the impacts of terrain effects on air pollution [48], particularly the basin effects [49,50]; some researchers have used geostatistical models to estimate the PM_2.5_ concentrations [51]. Fortunately, most of the tourist areas in Hsinchu County are located in areas with lower PM_2.5_ air pollution potentials, and the areas with higher air pollution potentials are mostly those with industrial and technological activities. Nevertheless, the areas with high pollution potentials have higher population densities. A high population density leads to more emission sources. Some researchers have used the spatial econometric model to investigate the relationship between the population density and air pollution in Chinese cities; they have discovered a significant positive correlation between the population density and PM_2.5_ concentration [52,53], which is consistent with the results of this study.

## 5. Conclusions

In this study, an air pollution potential map was constructed. The results show that the potentials of different seasons are quite different. The most severe air pollution seasons are spring and winter, whereas summer exhibits the best air quality. Xinfeng and Hukou Townships in Hsinchu County have the highest potential (approximately 18%). Hukou Old Street, which is the most famous tourist attraction, has a relatively high pollution risk. The population density is positively correlated with the PM_2.5_ potential distribution in most seasons, except for summer. In this study, the hazard potential levels of PM_2.5_ concentrations exceeding a certain standard were investigated; the exceedance probability and the air pollution potential levels of various tourist areas in Hsinchu County were examined. However, the information on tourist attractions considered in this research study is limited and based on only few important attractions. The air pollution potential map can be combined with more detailed tourist attraction maps in the future. In addition, the map can be applied to investigate the impacts of pollution on schools, elderly people, hospitals, and nurseries to determine their potential long-term exposure risks. Although the study area in Hsinchu County has only three important tourist attractions with the most severe air pollution potential levels (level 6), there are still many schools and residents in these areas.

In the future, a map for the entire country will be constructed; the proposed framework can be directly applied to other countries worldwide. In addition, the spatial and temporal changes in the air pollution potential during different years can be analyzed, and the air pollution data of one year can be expanded to more than five or ten years. In addition to reducing the possibility of being more extreme in certain years, understanding the temporal changes in the spatial distribution of the pollution potentials is more effective for assessing dynamic risks. In addition to providing a reference for tourists, the results provide information on the long-term health risks for local residents in the study area.

## Figures and Tables

**Figure 8:**
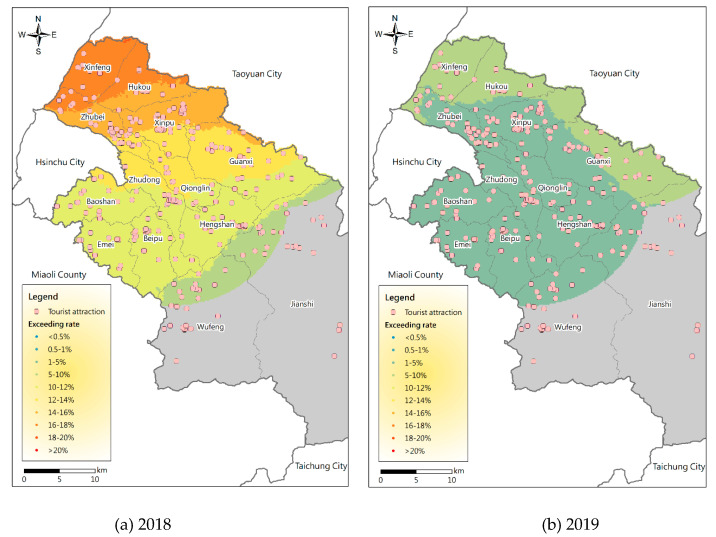
Dynamic distribution of PM_2.5_ potential in tourist areas in Hsinchu County in spring in (**a**) 2018 (**b**) 2019.

**Table 1:** Main industrial characteristics and potential disaster risks of 13 townships and cities in Hsinchu County.

District	Industry Type	Major Risk	Minor Risk	Other Risks
Zhubei City	Commerce and industry	Floods and droughts	Toxic chemicals	Tsunamis
Zhudong Township	Commerce and industry	Floods and droughts	Landslides/mudflows	
Hukou Township	Industry	Floods and droughts	Toxic chemicals	Air pollution
Baoshan Township	Industry	Landslides/mudflows	Toxic chemicals	Floods and droughts
Emei Township	Agriculture	Landslides/mudflows	Floods and droughts	
Wufeng Township	Agriculture	Landslides/mudflows	Floods and droughts	
Xinfeng Township	Agriculture and industry	Floods and droughts	Toxic chemicals	Air pollution or tsunamis
Qionglin Township	Agriculture and industry	Floods and droughts	Landslides/mudflows	Toxic chemicals
Beipu Township	Agriculture and industry	Landslides/mudflows	Floods and droughts	
Xinpu Township	Agriculture and industry	Floods and droughts	Landslides/mudflows	Toxic chemicals
Guanxi Township	Agriculture and leisure tourism	Floods and droughts	Landslides/mudflows	
Jianshi Township	Agriculture and leisure tourism	Landslides/mudflows	Floods and droughts	
Hengshan Township	Agriculture and leisure tourism	Landslides/mudflows	Floods and droughts	

**Table 2:** Basic statistics of exceedance probabilities of 13 townships and cities in Hsinchu County.

District	Entire Year	Spring	Summer	Fall	Winter
	Mean ± Standard Deviation (%)				
Zhubei City	7.6 ± 0.4	12.1 ± 0.8	0.1 ± 0.1	4.9 ± 0.6	13.2 ± 0.6
Zhudong Township	6.8 ± 0.3	11.4 ± 0.7	0.5 ± 0.3	4.4 ± 0.3	11.1 ± 0.6
Hukou Township	8.9 ± 0.6	16.8 ± 1.7	0.3 ± 0.1	5.8 ± 0.5	12.9 ± 0.3
Baoshan Township	6.5 ± 0.2	10.2 ± 0.4	0.2 ± 0.1	4.2 ± 0.3	11.7 ± 0.5
Emei Township	6.4 ± 0.1	9.9 ± 0.2	0.3 ± 0.1	4.5 ± 0.2	10.9 ± 0.4
Wufeng Township	5.9 ± 0.2	9.6 ± 0.4	0.4 ± 0.1	4.2 ± 0.2	9.5 ± 0.3
Xinfeng Township	9.0 ± 0.4	16.6 ± 1.5	0.2 ± 0.1	6.3 ± 0.4	13.1 ± 0.3
Qionglin Township	7.2 ± 0.1	12.1 ± 0.3	0.7 ± 0.2	4.6 ± 0.2	11.6 ± 0.3
Beipu Township	6.5 ± 0.3	10.6 ± 0.6	0.5 ± 0.1	4.5 ± 0.1	10.5 ± 0.4
Xinpu Township	7.7 ± 0.2	13.2 ± 0.7	0.3 ± 0.1	4.8 ± 0.4	12.5 ± 0.2
Guanxi Township	7.3 ± 0.4	11.8 ± 0.8	0.7 ± 0.1	5.0 ± 0.2	11.8 ± 0.5
Jianshi Township	6.2 ± 0.2	9.8 ± 0.4	0.5 ± 0.1	4.4 ± 0.3	10.1 ± 0.3
Hengshan Township	6.8 ± 0.2	11.2 ± 0.5	0.8 ± 0.1	4.6 ± 0.1	10.8 ± 0.3

**Table 3:** Levels of air pollution potential and numbers of affected tourist attractions.

Level of Air Pollution Potential	Exceedance Probability	Number of Tourist Attractions
0 (mild)	Below 5%	34
1	5% to 10%	34
2	10% to 12%	124
3	12% to 14%	114
4	14% to 16%	7
5	16% to 18%	11
6	18% to 20%	3
7 (severe)	More than 20%	0

**Table 4:** Highest air pollution potentials of tourist attractions—levels 5 and 6—in Hsinchu County.

Number	Name	District	Longitude	Latitude	Level of Air Pollution Potential
1	Caixiang Trail	Hukou Township	121.02028	24.891221	6
2	Xiansheng Temple	Hukou Township	121.047989	24.902892	6
3	Hukou Armored New Village (Village B)	Hukou Township	121.047808	24.904483	6
4	Rongyuanpu Farm	Hukou Township	121.0442	24.8754	5
5	Laohukou Catholic Church Cultural Center	Hukou Township	121.05516	24.87657	5
6	Renhe Trail	Hukou Township	121.058497	24.877032	5
7	Yao Art Street and Bicycle Taro	Hukou Township	121.0575	24.8773	5
8	Hanqing Trail	Hukou Township	121.05192	24.877399	5
9	Hukou Old Street	Hukou Township	121.052612	24.877742	5
10	Xinfeng Sanyuan Temple	Xinfeng Township	120.9979	24.8999	5
11	Yongning Temple	Xinfeng Township	120.985265	24.90248	5
12	Chifu Wangye Temple	Xinfeng Township	120.9764	24.9102	5
13	Hongmaogang Ecological Recreation Area	Xinfeng Township	120.976365	24.910229	5
14	Xinfengpuyuan Temple	Xinfeng Township	120.977599	24.924916	5

**Table 5:** Population density of each township in Hsinchu County in 2020.

District	Population Density (Persons/km^2^)
Zhubei City	3885.10
Zhudong Township	1811.10
Hukou Township	1325.41
Baoshan Township	224.58
Emei Township	118.33
Wufeng Township	20.02
Xinfeng Township	1226.25
Qionglin Township	491.86
Beipu Township	185.33
Xinpu Township	462.87
Guanxi Township	230.21
Jianshi Township	18.09
Hengshan Township	196.89

## Appendix A

**Table A1:** Detailed air pollution potential of each tourist attraction in Hsinchu County.

Number	Name	District	Longitude	Latitude	The Level of Air Pollution Potential
1	Caixiang trail	Hukou Township	121.02028	24.891221	6
2	Xiansheng Temple	Hukou Township	121.047989	24.902892	6
3	Hukou Armored New Village (Village B)	Hukou Township	121.047808	24.904483	6
4	Rongyuanpu Farm	Hukou Township	121.0442	24.8754	5
5	Laohukou Catholic Church Cultural Center	Hukou Township	121.05516	24.87657	5
6	Renhe Trail	Hukou Township	121.058497	24.877032	5
7	Yao Art Street and Bicycle Taro	Hukou Township	121.0575	24.8773	5
8	Hanqing Trail	Hukou Township	121.05192	24.877399	5
9	Hukou Old Street	Hukou Township	121.052612	24.877742	5
10	Xinfeng Sanyuan Temple	Xinfeng Township	120.9979	24.8999	5
11	Yongning Temple	Xinfeng Township	120.985265	24.90248	5
12	Chifu Wangye Temple	Xinfeng Township	120.9764	24.9102	5
13	Hongmaogang Ecological Recreation Area	Xinfeng Township	120.976365	24.910229	5
14	Xinfengpuyuan Temple	Xinfeng Township	120.977599	24.924916	5
15	Golden World Leisure Farm	Xinpu Township	121.022115	24.853193	4
16	Pinewood Brick and Tile Exhibition Hall	Xinfeng Township	120.990757	24.868983	4
17	Hukou Tourist Tea Garden	Hukou Township	121.0779	24.8729	4
18	Xinfeng Golf Course	Xinfeng Township	120.976496	24.882496	4
19	Zaixing Golf Course	Hukou Township	121.091008	24.883679	4
20	Xinfeng Wetland	Xinfeng Township	120.9719	24.9072	4
21	Xinfeng Seawall	Xinfeng Township	120.97	24.9075	4
22	Yuquanshan Puzhao Temple	Zhudong Township	121.082939	24.732835	3
23	Luliaokeng Trail	Qionglin Township	121.116898	24.733726	3
24	Forest Park Trail	Zhudong Township	121.084495	24.73457	3
25	Tree Qilin Cultural Center	Zhudong Township	121.095789	24.735623	3
26	Touqianxi Ecological Park	Zhudong Township	121.099787	24.736033	3
27	Five Harmony Temple	Qionglin Township	121.1201	24.7364	3
28	Zhudong Central Market	Zhudong Township	121.091482	24.736809	3
29	Ruanqiao Rainbow Village	Zhudong Township	121.091482	24.736809	3
30	Zhudong Forestry Exhibition Hall	Zhudong Township	121.093314	24.736851	3
31	Zhudong Forestry Exhibition Hall	Zhudong Township	121.093314	24.736851	3
32	Ue Pine Wood Bamboo East Branch Office	Zhudong Township	121.0932	24.7373	3
33	Draw a new page	Zhudong Township	121.094026	24.737928	3
34	Zhudong Railway Station	Zhudong Township	121.094831	24.738177	3
35	Zhudong City Bike Path	Zhudong Township	121.094742	24.738245	3
36	Flower World Play Cloth Workshop	Zhudong Township	121.08613	24.738522	3
37	Xiao Rusong Art Park	Zhudong Township	121.088201	24.739425	3
38	Xiao Rusong Former Residence Complex	Zhudong Township	121.088201	24.739425	3
39	Huangcheng Bamboo Curtain Cultural Center	Zhudong Township	121.091755	24.739675	3
40	Ganlu Temple	Zhudong Township	121.080823	24.744548	3
41	Juqing	Zhudong Township	121.0856	24.745265	3
42	Mingguan Art Museum	Zhudong Township	121.080119	24.745529	3
43	Duanmu Shiitake Mushroom Farm	Qionglin Township	121.140157	24.747489	3
44	Luliaokeng Mushroom Farm	Qionglin Township	121.1402	24.7475	3
45	Zhubei. Zhudongtou Qianxi Bicycle Path	Qionglin Township	121.094635	24.749005	3
46	Jiujiu Health Tomato Museum	Qionglin Township	121.095419	24.751802	3
47	Xionglin Luliaokeng Bell Room	Qionglin Township	121.140648	24.758295	3
48	Fulin Farm	Qionglin Township	121.090064	24.761744	3
49	Feifeng Wenchang	Qionglin Township	121.091294	24.762308	3
50	Shiming Tomato Farm	Guanxi Township	121.173188	24.765512	3
51	Wenlin Court	Qionglin Township	121.0826	24.7733	3
52	Deng Yuxian Music and Culture Memorial Park	Qionglin Township	121.085126	24.773326	3
53	Zhiliaowo Papermaking Workshop	Qionglin Township	121.082624	24.780282	3
54	Jin Yong DIY Tomato Farm	Guanxi Township	121.180745	24.782149	3
55	Jin Guangfu Mansion	Guanxi Township	121.176841	24.78657	3
56	Luo Wu College	Guanxi Township	121.175658	24.787931	3
57	Guanxi Windward Museum	Guanxi Township	121.183708	24.788557	3
58	Guanxi Taiwan Black Tea Company	Guanxi Township	121.175753	24.791305	3
59	Taiwan Red Tea Cultural Center	Guanxi Township	121.175753	24.791305	3
60	Guanxi Donganqiao	Guanxi Township	121.178174	24.791512	3
61	Instant burned grass, natural ancient flavor [Agricultural good companion 1. Guanxi Town Farmers’ Association Tour]	Guanxi Township	121.176829	24.791634	3
62	Guanxi Niulan River Bicycle Path	Guanxi Township	121.180862	24.792248	3
63	Guanxi Catholic Church	Guanxi Township	121.176419	24.794329	3
64	Xinbao Tourist Orchard	Xinpu Township	121.085477	24.796986	3
65	Pinglin Hiking Trail	Guanxi Township	121.14066	24.80115	3
66	Mingdeng Ancient Road	Guanxi Township	121.187	24.802	3
67	Guanxi Town Farmers’ Association Xiancao Processing Factory	Guanxi Township	121.162535	24.8029	3
68	Yuanhe Temple	Guanxi Township	121.135645	24.803515	3
69	Daluo Strawberry Farm	Guanxi Township	121.160606	24.803821	3
70	Fukuda Strawberry Farm	Guanxi Township	121.160099	24.804235	3
71	Gaoping Tomato Farm	Guanxi Township	121.152527	24.805734	3
72	Gillian Strawberry Farm	Guanxi Township	121.144999	24.805949	3
73	Lu Ji Farm	Guanxi Township	121.144999	24.805949	3
74	Shiquan Farm	Guanxi Township	121.144999	24.805949	3
75	Da Asah Valley Orchid Farm	Guanxi Township	121.140345	24.809045	3
76	Xiangzhangyuan Leisure Farm	Xinpu Township	121.080152	24.810143	3
77	Agen Strawberry Farm	Guanxi Township	121.116085	24.816814	3
78	Shuangyuan Leisure Farm	Zhubei City	121.0357	24.8239	3
79	Leofoo Village Theme Park	Guanxi Township	121.180728	24.824679	3
80	Yunhai Leisure Tea Factory	Guanxi Township	121.162268	24.824891	3
81	Xiaolixi Bicycle Path	Xinpu Township	121.087924	24.825068	3
82	Yuanxin Persimmon	Guanxi Township	121.1168	24.8254	3
83	Guannanyangtang Tang House	Guanxi Township	121.114123	24.82637	3
84	Xinpu Liu Family Ancestral Hall	Xinpu Township	121.075093	24.827271	3
85	Xinpu Zhu Family Temple	Xinpu Township	121.076351	24.827356	3
86	Xinpu Pan House	Xinpu Township	121.075982	24.827584	3
87	Sky, People, Things, I-Whole People Xinpu	Xinpu Township	121.071	24.828	3
88	Zhaomen Agricultural Recreation Area	Xinpu Township	121.071278	24.828042	3
89	Xinpu Elementary School Principal Dormitory	Xinpu Township	121.079223	24.828081	3
90	Happy childhood	Xinpu Township	121.079138	24.828126	3
91	Zhu Jincheng Studio	Xinpu Township	121.036754	24.82826	3
92	Wow, delicious persimmon!	Xinpu Township	121.074935	24.828382	3
93	Xinpu Chen’s Ancestral Hall	Xinpu Township	121.076451	24.828393	3
94	Xinpu Fan Family Temple	Xinpu Township	121.07597	24.828541	3
95	Xinpu Lin Family Temple	Xinpu Township	121.0761	24.8292	3
96	Comic Art Square	Xinpu Township	121.073196	24.829343	3
97	Yiyuan Hakka Cuisine	Guanxi Township	121.122728	24.830801	3
98	New farmers market	Xinpu Township	121.087698	24.831114	3
99	Sansheng Temple	Xinpu Township	121.098799	24.831432	3
100	Flying Dragon Hiking Trail	Xinpu Township	121.098799	24.831432	3
101	Persimmon Dyeing Workshop	Xinpu Township	121.079142	24.833894	3
102	Zhubei Tianhou Temple	Zhubei City	121.011231	24.835694	3
103	Shaotanwo Old Road	Xinpu Township	121.049186	24.837801	3
104	Wu Zhuoliu’s Former Residence	Xinpu Township	121.109831	24.838011	3
105	Xinpu Shangfangliao Liu House	Xinpu Township	121.04949	24.838082	3
106	Chunhe Farm	Xinpu Township	121.039527	24.838179	3
107	The happy persimmon feeling blown by the wind	Xinpu Township	121.076663	24.840959	3
108	Barbarian’s Fortune Land	Zhubei City	120.997423	24.841493	3
109	Fengshanxi Fangliao Village Bicycle Path	Xinpu Township	121.0442	24.8416	3
110	Zhubei Citizen Farm	Zhubei City	120.998011	24.842051	3
111	Xinpu Baozhong Pavilion	Xinpu Township	121.036271	24.843354	3
112	Jinhan Dried Persimmons, Arrow Bamboo Nest, Orchard, Zhulan Garden” Rural Regeneration Tour of Daping Community, Xinpu 1	Xinpu Township	121.078579	24.844258	3
113	Drying Persimmon in Jinhan Farm	Xinpu Township	121.078578	24.84426	3
114	Shangpinxiang Orchard	Xinpu Township	121.069021	24.84437	3
115	Li Village Farm	Xinpu Township	121.0715	24.84738	3
116	Zhaomen Trail Group-Huaizu Trail	Xinpu Township	121.105264	24.848389	3
117	Fuming New Farm	Xinpu Township	121.101378	24.849639	3
118	Lin Family Orchard	Xinpu Township	121.101759	24.851476	3
119	Gou Bei Kiln Studio	Zhubei City	120.985133	24.852155	3
120	Nanping, Beipingli Bicycle Path	Xinpu Township	121.0864	24.8525	3
121	Bamboo Garden	Xinpu Township	121.101963	24.852972	3
122	Crossing the Borders and Traveling in the North Country Scenery ~ Winter’s Jingu Farm	Xinpu Township	121.105574	24.854441	3
123	Fuxiang Cactus Succulent Botanical Garden	Xinpu Township	121.092191	24.855623	3
124	Liujiazhuang Braised Chicken	Xinpu Township	121.105662	24.856742	3
125	Zhoujiazhuang Sightseeing Farm (Recreation Inn)	Xinpu Township	121.1042	24.857374	3
126	Red Dragon Fruit Sightseeing Orchard	Zhubei City	120.96128	24.858706	3
127	Chenjia Farm	Xinpu Township	121.103731	24.860894	3
128	Zhaomen Trail Group-Guannan Trail	Xinpu Township	121.1037	24.8609	3
129	Wind movement, Jinghai, Xiange	Zhubei City	120.963966	24.861473	3
130	Zhubei‧Binhai Recreation Area	Zhubei City	120.946333	24.865234	3
131	Tiande Temple	Xinfeng Township	120.9797	24.8675	3
132	Zhubei Coastal Forest Conservation Area	Zhubei City	120.95143	24.87045	3
133	Lianhua Temple	Zhubei City	120.961164	24.876555	3
134	Fengqi Sunset	Zhubei City	120.961164	24.876555	3
135	Zhubei Lotus Temple Wetland	Zhubei City	120.9612	24.8766	3
136	Sakura Forest Leisure Farm	Wufeng Township	121.092207	24.63246	2
137	Liangshan Tribe	Wufeng Township	121.1236	24.6451	2
138	Shangrui Orange Garden	Zhudong Township	121.1151	24.6626	2
139	Beipu Cold Spring	Beipu Township	121.072811	24.663056	2
140	Shangping Old Street	Zhudong Township	121.093986	24.66359	2
141	Youdian Grass Ecological Farm	Beipu Township	121.05329	24.67397	2
142	Huisen Natural Leisure Farm	Beipu Township	121.0545	24.6744	2
143	Riding a Dragon	Hengshan Township	121.150594	24.68251	2
144	Dashanbei Leisure Farm	Hengshan Township	121.150594	24.68251	2
145	Emei Catholic Church	Emei Township	121.021722	24.688162	2
146	Emei Catholic Church	Emei Township	121.021722	24.688162	2
147	Mingsheng Ecological Leisure Farm	Beipu Township	121.041911	24.688309	2
148	Emei Lake Scenic Area	Emei Township	121.019586	24.688769	2
149	Dangui Temple	Emei Township	121.021527	24.688846	2
150	Emei Elementary School	Emei Township	121.020109	24.688953	2
151	Dahu Mountain Forest	Beipu Township	121.087718	24.690279	2
152	Bamboo Yucha Reed Sweet Potato	Beipu Township	121.041675	24.692211	2
153	Summer Garden Organic Farm	Zhudong Township	121.102428	24.69276	2
154	King Kong Temple	Beipu Township	121.044	24.6928	2
155	North Point Suspension Bridge	Jianshi Township	121.202652	24.696684	2
156	Maike Tianyuan Leisure Farm	Beipu Township	121.042268	24.697066	2
157	Beipu Jiang Family Temple	Beipu Township	121.056501	24.697733	2
158	Xiaomi decorative artwork	Jianshi Township	121.205046	24.698166	2
159	Deng Nanguang Image Memorial Hall	Beipu Township	121.058038	24.698537	2
160	Deng Nanguang Image Memorial Hall	Beipu Township	121.058038	24.698537	2
161	Beipu Zhongshu Church	Beipu Township	121.057879	24.698851	2
162	Dashanbei Leshantang	Hengshan Township	121.139447	24.699327	2
163	Chen Yongbin Woodworking DIY Studio	Beipu Township	121.04361	24.699473	2
164	Xiuluan Park	Beipu Township	121.0601	24.6996	2
165	Beipu Old Street, Nanpu Village Bicycle Path	Beipu Township	121.057392	24.6997	2
166	Green World Leisure Farm	Beipu Township	121.072648	24.699712	2
167	Beipu Citian Temple	Beipu Township	121.058449	24.699739	2
168	Beipu Township “Farmers Direct Sales Station”	Beipu Township	121.055402	24.70079	2
169	Erliao Shenmu	Beipu Township	121.056389	24.702038	2
170	Wuzhi Shan Scenic Area	Beipu Township	121.056389	24.702038	2
171	Neiwan Old Street	Hengshan Township	121.1322	24.7025	2
172	Sharing and glory	Jianshi Township	121.199393	24.70343	2
173	Huazhouyuan Puppet Theater	Hengshan Township	121.180842	24.704501	2
174	Jianshiyan	Jianshi Township	121.201251	24.705095	2
175	Da Ba Jianshan	Jianshi Township	121.201251	24.705095	2
176	Aboriginal Cultural Relics Museum of Jianshi Township	Jianshi Township	121.201251	24.705095	2
177	Neiwan Station	Hengshan Township	121.182277	24.705331	2
178	Xiaojiao’s Cheering Paradise	Hengshan Township	121.182277	24.705331	2
179	Riverbank Hot Springs	Hengshan Township	121.175728	24.705483	2
180	Water Moon Bay Wonderland	Hengshan Township	121.180002	24.705915	2
181	Neiwan Police Station	Hengshan Township	121.182453	24.706254	2
182	Neiwan Catholic Church	Hengshan Township	121.18067	24.706336	2
183	Guangji Temple	Hengshan Township	121.181782	24.706458	2
184	Jack and the Magic Bean	Hengshan Township	121.169889	24.706619	2
185	Inner Bay Suspension Bridge	Hengshan Township	121.180469	24.706837	2
186	Ancient Trojan Horse Road	Hengshan Township	121.183028	24.707095	2
187	Tenren Rock House	Hengshan Township	121.1665	24.7105	2
188	Toyota Village, Baishi Lake Bicycle Path	Hengshan Township	121.166472	24.710547	2
189	Watermelon Manor Cultural Education Park	Beipu Township	121.059063	24.715161	2
190	Watermelon Manor	Beipu Township	121.059063	24.715161	2
191	Fengxiang Waterfall Recreation Area	Hengshan Township	121.142277	24.715778	2
192	Youluo Valley	Hengshan Township	121.142277	24.715778	2
193	Hexin, Hexing, everyone agrees	Hengshan Township	121.15353	24.716795	2
194	Hexing Station	Hengshan Township	121.15353	24.716795	2
195	Fugui Station	Hengshan Township	121.15346	24.717244	2
196	Inspiration Pumping Truck	Hengshan Township	121.121782	24.717573	2
197	Cihuitang	Zhudong Township	121.074723	24.721329	2
198	Boss Leisure Farm	Hengshan Township	121.131424	24.726749	2
199	Shishang Hot Spring	Jianshi Township	121.222791	24.730172	2
200	Baoshan Golf Course	Baoshan Township	120.943582	24.73083	2
201	Wax Candle Art House	Baoshan Township	120.960506	24.730999	2
202	Jianshih Lavender Cottage	Jianshi Township	121.233957	24.733288	2
203	Fusha Osaki Trail	Hengshan Township	121.1658	24.735299	2
204	Petite Teresa Church	Baoshan Township	120.9689	24.7356	2
205	Baoshan Sugar Factory Bicycle Road Line	Baoshan Township	120.970236	24.735987	2
206	Wetland farm	Qionglin Township	121.14539	24.736695	2
207	Songtao Tianyuan Leisure Farm	Baoshan Township	121.020534	24.736961	2
208	Baoshan Reservoir and Baoshan Second Reservoir	Baoshan Township	121.038856	24.738962	2
209	Nine Dragon Temple	Baoshan Township	120.974491	24.747297	2
210	Xuyang Golf Course	Guanxi Township	121.183553	24.747565	2
211	Shahuli Art Village	Baoshan Township	121.044635	24.750122	2
212	Lord Guanxi Golf Course	Guanxi Township	121.197329	24.752341	2
213	Blonde Pitaya Farm	Guanxi Township	121.169523	24.752877	2
214	Double-vitality-hope	Zhudong Township	121.055347	24.765024	2
215	Goyulang Tribe	Guanxi Township	121.241998	24.766027	2
216	Huashan Leisure Farm	Guanxi Township	121.178748	24.766915	2
217	Mountain Creek Golf Course	Guanxi Township	121.211636	24.767379	2
218	Zhudong Dazhen	Zhudong Township	121.056815	24.767488	2
219	Jin Geum Shan Yimin Temple	Guanxi Township	121.22436	24.767702	2
220	Guanxi Bat Cave	Guanxi Township	121.224211	24.767959	2
221	Shenjing Village Tea Garden District	Baoshan Township	120.999394	24.76848	2
222	Baohu Suspension Bridge. Bihu Suspension Bridge	Baoshan Township	120.999394	24.76848	2
223	Geumsan Shiitake Farm	Guanxi Township	121.229277	24.770571	2
224	Two monuments at Zhudongtou	Zhudong Township	121.029867	24.780819	2
225	Sleepy bear	Zhudong Township	121.030683	24.781257	2
226	Li Yi Golf Course	Guanxi Township	121.190366	24.783718	2
227	Jin Guangcheng Cultural Center	Guanxi Township	121.212456	24.788457	2
228	Xionglin. Six bicycle lanes	Qionglin Township	121.074692	24.790157	2
229	Shiniu Mountain Trail	Guanxi Township	121.253322	24.793516	2
230	Mercy Farm	Guanxi Township	121.231258	24.798199	2
231	Lonely Odoby	Zhubei City	121.03933	24.807568	2
232	The birth of new Gila	Zhubei City	121.03933	24.807568	2
233	Hsinchu High Speed Rail Station	Zhubei City	121.040226	24.808196	2
234	Zhubei Tongdetang	Zhubei City	121.047616	24.809173	2
235	Zhubei Liuzhanglilin Family Shrine	Zhubei City	121.0222	24.8107	2
236	Zhubei Six Zhangli Doctor	Zhubei City	121.02444	24.810887	2
237	Four-sided view	Zhubei City	121.035146	24.811017	2
238	Xinwawu Hakka Culture Preservation Area	Zhubei City	121.026943	24.811667	2
239	Zhubei Liuzhangli Zhongxiao Hall (No. 13 Dongpingli)	Zhubei City	121.025204	24.811753	2
240	Zhubei Liuzhangli asked the auditorium	Zhubei City	121.02511	24.811791	2
241	Chubei Quanzhou Chuo Fenyang Hall	Zhubei City	121.017008	24.816685	2
242	Bodhi Love	Zhubei City	121.031998	24.820934	2
243	Zhubei Stadium	Zhubei City	121.022673	24.821273	2
244	Litou Mountain Trail	Xinpu Township	121.045586	24.821301	2
245	Zhubei Liuzhangli Zhongxiao Hall (No. 18, Dongpingli)	Zhubei City	121.0142	24.8221	2
246	Zhubei County Fuyuan	Zhubei City	121.015146	24.824672	2
247	Lianhua Temple	Zhubei City	121.025643	24.825271	2
248	Zhubei Lianhua Temple	Zhubei City	121.025643	24.825271	2
249	Time story	Zhubei City	121.01073	24.826267	2
250	Hsinchu County Government	Zhubei City	121.0129	24.8269	2
251	Zhubei Guangming Commercial District	Zhubei City	121.019572	24.828918	2
252	Collection, Fenghua	Zhubei City	121.012496	24.830096	2
253	Hsinchu County Art Museum	Zhubei City	121.012496	24.830096	2
254	Hsinchu County History Museum	Zhubei City	121.012496	24.830096	2
255	Hsinchu County History Museum	Zhubei City	121.012496	24.830096	2
256	Dingfeng Bee Farm	Zhubei City	120.992908	24.833797	2
257	Li Longquan Multi-art Space	Zhubei City	120.986656	24.836262	2
258	Niupu Creek‧Mangrove Scenic Area	Zhubei City	120.948543	24.851247	2
259	Tokai Organic Lime Garden	Zhubei City	120.947401	24.853197	2
260	Guize Mountain Trail	Wufeng Township	121.123057	24.614147	1
261	Wufeng Liangshan Camping Area	Wufeng Township	121.102357	24.615732	1
262	Saixia Basdaai Festival	Wufeng Township	121.0994	24.6225	1
263	Guyan Waterfall	Wufeng Township	121.12403	24.624802	1
264	Bamboo Forest Health Village Cooperative	Wufeng Township	121.120559	24.625633	1
265	Maibari tribe	Wufeng Township	121.120672	24.625933	1
266	Fairy Lake Camping Area	Wufeng Township	121.116313	24.626549	1
267	Shengying Farm and Aboriginal Rattan Weaving	Wufeng Township	121.143845	24.631032	1
268	Qingquan Scenery Area	Wufeng Township	121.119632	24.632065	1
269	Bailan Tribe	Wufeng Township	121.119632	24.632065	1
270	Heping Tribe Recreational Agriculture Area	Wufeng Township	121.119632	24.632065	1
271	Saixia Dwarf Spirit Festival	Wufeng Township	121.119632	24.632065	1
272	Meihouman Waterfall	Wufeng Township	121.157475	24.649665	1
273	Wan Fo An	Emei Township	121.02287	24.65199	1
274	Shuilian Bridge Trail	Emei Township	121.024447	24.655557	1
275	Lion Mountain Trail	Emei Township	121.024447	24.655557	1
276	Tianhu Farm	Jianshi Township	121.179965	24.668714	1
277	Song Yunxuan Coffee House	Emei Township	120.991693	24.668766	1
278	Plum Blossom Villa	Jianshi Township	121.195189	24.674083	1
279	Shiliiao Leisure Agricultural Park	Emei Township	120.986031	24.675063	1
280	Emei Lake, Twelve Liao, Shishan Visitor Center Bicycle Path	Emei Township	120.985319	24.6794	1
281	Little Raindrop Art Space	Emei Township	120.974407	24.688101	1
282	Emei Fuxing Tea Factory (including the House of Lu Kingdom and Zeng Zhengzhang)	Emei Township	120.971711	24.688161	1
283	Shen Dongning Studio	Emei Township	121.0094	24.6909	1
284	Fuxing Tea Exhibition Center	Emei Township	120.986019	24.69716	1
285	Dance of Youth	Emei Township	120.998063	24.715319	1
286	Fengcheng Charcoal Kiln (House of Charcoal)	Baoshan Township	120.997016	24.721236	1
287	Dongkeng Xinfeng Temple	Baoshan Township	120.980247	24.722726	1
288	Sanfeng Farmers’ Orchard	Baoshan Township	120.997522	24.724663	1
289	Dongkeng Bogong Temple	Baoshan Township	120.985804	24.731756	1
290	Nun temple	Baoshan Township	120.977819	24.750516	1
291	Baosheng Temple	Baoshan Township	121.009258	24.750518	1
292	Sunfull Temple	Baoshan Township	120.990303	24.765395	1
293	Baoshan Ecological Farm Pond	Baoshan Township	120.991274	24.765525	1
294	Baxian Waterfall	Wufeng Township	121.095289	24.534444	0
295	Cinsbus Giant Trees	Jianshi Township	121.296087	24.54063	0
296	Town West Fort Church	Jianshi Township	121.3024	24.5731	0
297	Huang Guanglai Greenhouse Honey Peach Garden (Duanmu Mushroom Garden)	Jianshi Township	121.301585	24.573782	0
298	Sanmao Residence	Wufeng Township	121.105808	24.573931	0
299	Qingquan Hot Spring	Wufeng Township	121.105564	24.574473	0
300	Taoshan Elementary School	Wufeng Township	121.106182	24.57514	0
301	Leha Mountain Farm Camping Area	Wufeng Township	121.0799	24.5753	0
302	Guanwu National Forest Recreation Area	Wufeng Township	121.113756	24.575489	0
303	Qingquan Catholic Church	Wufeng Township	121.10381	24.576976	0
304	Yuanyang Lake Natural Ecological Conservation Area	Jianshi Township	121.406221	24.577652	0
305	People have sculpture park	Wufeng Township	121.107493	24.579401	0
306	Bailan Leisure Agriculture Area	Wufeng Township	121.087456	24.579457	0
307	Xinguang Tribe	Jianshi Township	121.3032	24.5799	0
308	Xiweng Waterfall	Wufeng Township	121.1481323	24.5915394	0
309	Taoshan Tunnel	Wufeng Township	121.108272	24.600923	0
310	Tianyue Farm	Wufeng Township	121.095966	24.603535	0
311	Shanshang Renjia Leisure Farm	Wufeng Township	121.089037	24.604624	0
312	Liying Mountain Trail	Jianshi Township	121.3338	24.6526	0
313	Jianshi TAPUNG Castle (Li Wei Aiyong Supervision Office)	Jianshi Township	121.322805	24.660641	0
314	Jinmei Suspension Bridge	Jianshi Township	121.207775	24.670304	0
315	Natural Valley Hot Spring	Jianshi Township	121.2696	24.6718	0
316	Secret Garden Coffee Garden	Jianshi Township	121.251724	24.677793	0
317	Shanqing Leisure Farm	Jianshi Township	121.21037	24.678877	0
318	Naluowan Leisure Farm	Jianshi Township	121.243623	24.679272	0
319	Luoxing Trout Leisure Farm	Jianshi Township	121.236604	24.679805	0
320	Hengshan and Ulao Bicycle Paths	Jianshi Township	121.247229	24.680325	0
321	Jinping Church	Jianshi Township	121.2287	24.6977	0
322	Jinping Park	Jianshi Township	121.218639	24.698443	0
323	Linghai Mountain Forest Leisure Farm	Jianshi Township	121.2831	24.7065	0
324	Bu Lao Ju Leisure Farm	Jianshi Township	121.2693	24.7135	0
325	Lao Liu Orchard in Bawu Mountain	Jianshi Township	121.279338	24.716784	0
326	Bali Forest Hot Spring Resort	Jianshi Township	121.235676	24.721937	0
327	Paddy field camp	Jianshi Township	121.259345	24.734987	0
